# Macrophage galactose-type lectin (MGL) is induced on M2 microglia and participates in the resolution phase of autoimmune neuroinflammation

**DOI:** 10.1186/s12974-019-1522-4

**Published:** 2019-06-27

**Authors:** Juan M. Ilarregui, Gijs Kooij, Ernesto Rodríguez, Susanne M. A. van der Pol, Nathalie Koning, Hakan Kalay, Joost C. van der Horst, Sandra J. van Vliet, Juan J. García-Vallejo, Helga E. de Vries, Yvette van Kooyk

**Affiliations:** 1 Department of Molecular Cell Biology and Immunology, Amsterdam Infection and Immunity Institute, Amsterdam UMC, Vrije Universiteit Amsterdam, O|2 building, room 11 E 41, PO Box 7057, Amsterdam, 1007MB Noord-Holland The Netherlands; 2 Department of Molecular Cell Biology and Immunology, Amsterdam Neuroscience, VUmc MS Center, Amsterdam UMC, Vrije Universiteit Amsterdam, Amsterdam, the Netherlands

**Keywords:** C-type lectin receptors, MGL, Multiple sclerosis, Experimental autoimmune encephalomyelitis, Microglia, Inflammation, Tolerance

## Abstract

**Background:**

Multiple sclerosis (MS) involves a misdirected immune attack against myelin in the brain and spinal cord, leading to profound neuroinflammation and neurodegeneration. While the mechanisms of disease pathogenesis have been widely studied, the suppression mechanisms that lead to the resolution of the autoimmune response are still poorly understood. Here, we investigated the role of the C-type lectin receptor macrophage galactose-type lectin (MGL), usually expressed on tolerogenic antigen-presenting cells (APCs), as a negative regulator of autoimmune-driven neuroinflammation.

**Methods:**

We used in silico, immunohistochemical, immunofluorescence, quantitative real-time polymerase chain reaction (qRT-PCR) and flow cytometry analysis to explore the expression and functionality of MGL in human macrophages and microglia, as well as in MS post-mortem tissue. In vitro, we studied the capacity of MGL to mediate apoptosis of experimental autoimmune encephalomyelitis (EAE)-derived T cells and mouse CD4^+^ T cells. Finally, we evaluated in vivo and ex vivo the immunomodulatory potential of MGL in EAE.

**Results:**

MGL plays a critical role in the resolution phase of EAE as MGL1-deficient (*Clec10a*^−/−^) mice showed a similar day of onset but experienced a higher clinical score to that of WT littermates. We demonstrate that the mouse ortholog MGL1 induces apoptosis of autoreactive T cells and diminishes the expression of pro-inflammatory cytokines and inflammatory autoantibodies. Moreover, we show that MGL1 but not MGL2 induces apoptosis of activated mouse CD4^+^ T cells in vitro. In human settings, we show that MGL expression is increased in active MS lesions and on alternatively activated microglia and macrophages which, in turn, induces the secretion of the immunoregulatory cytokine IL-10, underscoring the clinical relevance of this lectin.

**Conclusions:**

Our results show a new role of MGL-expressing APCs as an anti-inflammatory mechanism in autoimmune neuroinflammation by dampening pathogenic T and B cell responses, uncovering a novel clue for neuroprotective therapeutic strategies with relevance for in MS clinical applications.

**Electronic supplementary material:**

The online version of this article (10.1186/s12974-019-1522-4) contains supplementary material, which is available to authorized users.

## Background

Multiple sclerosis (MS) is a neurological autoimmune disorder of the central nervous system (CNS), characterized by recurrent episodes of inflammatory demyelination, leading to a progressive deterioration of the neurological functions [1]. Although recent studies have revealed that both innate and adaptive immune cells contribute to the pathogenesis and progression of MS and its in vivo model experimental autoimmune encephalomyelitis (EAE) [2], there is little information about the mechanisms involved in the resolution of inflammation during remission, a process that leads to clinical improvement of patients. Besides the dampening role of IL-10- and TGF-β-producing regulatory cells [3], just a handful of mechanisms have been associated with the resolution of the autoimmune neuroinflammation. Recent evidence has linked cytotoxic T-lymphocyte antigen-4 (CTLA-4), programmed death-1 (PD-1), T cell immunoreceptor with immunoglobulin and immunoreceptor tyrosine-based inhibition motif domains (TIGIT), and T cell immunoglobulin- and mucin domain-containing molecule 3 (TIM-3) as important immune regulatory pathways in the resolution of EAE, showing a strong genetic or functional correlation in MS (reviewed in [4]). Interestingly, all the depicted pathways involve the inhibition of the activity of myelin-reactive T cells.

The macrophage galactose-type lectin (MGL; aka Clec10a or CD301) is a member of the C-type lectin receptor (CLR) family [5, 6]. This family is composed of a variety of transmembrane and soluble receptors that recognize glycan structures in a Ca^2+^-dependent manner through a common carbohydrate recognition domain (CRD) and play important roles in both innate and adaptive immune responses [6]. MGL is expressed by myeloid antigen-presenting cells (APC) such as dendritic cells (DCs) and macrophages [5]. Human MGL and its mouse ortholog MGL2 bind *O*-glycans decorated with sTn or Tn antigens (sialylated and non-sialylated *N*-acetylgalactosamine, αGalNAc), residues, and the LacdiNAc epitope (GalNAcβ1-4GlcNAc). In contrast, the mouse ortholog MGL1 binds Lewis-A and Lewis-X structures [5, 7].

MGL binding to CD45 on activated CD4^+^ T cells mediates effector T cell apoptosis, leading to the dampening of the inflammatory response [8]; while in the APC compartment, MGL triggering induces IL-10 production and inhibits cell maturation and migration [9–14]. It has been proposed that these effects are exploited by pathogens and tumors as immune escape mechanisms [5, 7, 11, 13, 15, 16]. At the same time, the immunoregulatory properties of MGL are involved in dampening autoimmune diseases in different settings [5, 10]. However, its role in autoimmune neuroinflammation has not been reported.

Here, we present data showing that MGL1, but not MGL2, participates in the resolution phase of the disease by inducing apoptosis of autoreactive T cells and diminishing the expression of pro-inflammatory cytokines and autoantibodies. Moreover, in human settings, we show that MGL expression is increased in active MS lesions and on alternatively activated microglia and macrophages which, in turn, induces the secretion of the immunoregulatory cytokine IL-10. Altogether, these data demonstrate that MGL acts as a negative regulator in autoimmune-induced neuroinflammation, uncovering a novel clue for neuroprotective therapeutic strategies with relevance in MS clinical applications.

## Methods

### Mice

MGL1-deficient mice (*Clec10a*^−/−^; C57BL/6) [17] were provided by the Consortium for Functional Glycomics (www.functionalglycomics.org). C57BL/6 mice were purchased from Charles River Laboratories (Maastricht, The Netherlands). Mice were bred and housed at the Amsterdam Animal Research Center (VU/VUmc) under specific pathogen-free conditions. All experiments were approved by the Animal Experiments Committee of the VUmc and performed in accordance with the national and international guidelines and regulations.

### Reagents

MGL1 and MGL2 were generated by fusing the extracellular domain of each lectin to a human IgG1-Fc tail as described [7], fluorescein isothiocyanate (FITC)-annexin V staining (BD Biosciences), unlabeled anti-MGL (clones 18E4 and 1G6.6 as described [8]), phycoerythrin (PE)-anti-MGL (Miltenyi, REA586), anti-mMHC II (clone LN3, kind gift from Amsterdam UMC, location VUmc Amsterdam, department of pathology, the Netherlands), and anti-P2Y12R (Anaspec).

### Generation 2 cystamine core Gal and GalNac dendrimers

Gal-Gal or GalNac-Gal (Elicityl) were conjugated to generation 2 cystamine core PAMAM dendrimers (Sigma-Aldrich) via reductive amination. Briefly, 1 equivalent of dendrimer was mixed with 40 equivalents of glycan in 200 μl acetic acid-dimethyl sulfoxide mixture (20% and 80%, respectively, both from Sigma-Aldrich). After thoroughly mixing, 80 equivalents of 2-picoline-borane complex (Sigma-Aldrich) was added and incubated at 65 °C for 3 h. For dendrimer glycation, a mixture of dichloromethane-diethyl ether (1:1, both from Sigma-Aldrich) was added and thoroughly vortexed. Glycated dendrimers were pelleted by centrifugation at 14000 rpm for 1 min. The supernatant was discarded, and the pellet was washed 3 times with diethyl ether (Sigma-Aldrich) and pelleted again by centrifugation. Pellet was dissolved in MilliQ water and lyophilized. Then, lyophilized glycated dendrimers were dissolved and purified over Superdex 200 10/300 GL column (GE Healthcare Life Sciences) on an Ultimate 3000 HPLC system (ThermoFisher).

### Mouse T cells

Naïve CD4+ T cells (CD62L+ CD44low) were isolated from mouse spleens using the naive CD4^+^ T Cell Isolation Kit (Miltenyi Biotec) according to the manufacturer’s protocol and stimulated (2 × 10^6^ cells/ml) with plate-bound anti-CD3 (3 μg/ml; clone 145-2C11; BD Biosciences) and soluble anti-CD28 (1 μg/ml; clone 37.51; BD Biosciences) for 3 days.

### Cell death assays

Activated CD4^+^ T cells (1 × 10^6^ cells/ml) were incubated with 10 μg/ml MGL1− and/or MGL2-Fc in RPMI for different time periods. Apoptotic cells were identified by AV/7-AAD staining. Cell death was determined as the difference in the percentage of AV and 7-AAD double-negative cells between polarized cells treated with or without each stimulus. For ex vivo experiments with cells from mice with EAE, draining lymph node (DLN) cells were stimulated ON in the presence or absence of 10 μg/ml MGL1− and/or MGL2-Fc in RPMI 10% FCS. Then, cells were stained and analyzed as stated above.

### Induction and assessment of EAE in mice

EAE was induced in 8–12-week-old female *Clec10a*^−/−^ and wild-type mice (C57BL/6) by s.c. immunization with 150 μg mouse MOG_35–55_ (MEVGWYRSPFSRVVHLYRNGK; Cambridge Research Biochemicals) in complete Freund’s adjuvant (CFA) supplemented with 4 mg/ml *Mycobacterium tuberculosis* (H37RA; Difco). Control animals were injected with a 1:1 PBS/CFA mixture. All animals received 200 ng pertussis toxin (Sigma) i.p. on days 0 and 2. Mice were examined daily for signs of EAE and scored as follows: 0, no clinical signs; 0.5, half limp tail; 1, complete limp tail; 1.5, lack of toe-spreading reflex; 2, half hind limb weakness; 2.5, hind limb weakness; 3, half hind limb paralysis; 3.5, incomplete hind limb paralysis; 4, complete hind limb paralysis; 4.5, diaphragmatic paralysis/paralysis of (one of the) front legs; and 5, death by EAE. At day 27, proliferation was determined in antigen-specific splenocytes and draining lymph node (DLN) cells by [^3^H]-thymidine incorporation following ex vivo restimulation with 25 μg/ml MOG_35–55_. Cytokine production was determined in supernatants following 72-h antigen restimulation by ELISA. The IL-17 ELISA kit was from R&D.

### Rat acute EAE

We used EAE data acquired from an independent study performed in our laboratory, and the acute EAE was induced as described previously [18]. For microarray analysis, 1 μg of total RNA was linearly amplified (at ServiceXS) by T7 RNA amplification, and Cy3 or Cy5 was incorporated during the cDNA synthesis according to the manufacturer’s instructions (Agilent Technologies). Equal amounts of Cy3- and Cy5-labeled samples were hybridized 17 h on a rat Agilent oligo microarray. For the cerebellum, samples of two EAE animals and two CFA control animals per time point were hybridized separately in a loop-style experimental setup, using four microarrays per time point. Because of the small sample size, the samples of the brainstem of two animals per time point were pooled after RNA isolation and hybridized in a direct dye swap, using two microarrays per time point. The arrays were scanned with an Agilent G2565AA dual-laser microarray scanner. The resulting images were analyzed with the Agilent Feature Extraction Software (www.agilent.com). In brief, in the first step, outliers were detected, then the values were corrected for background and normalized using the linear/Lowess method as described in the Agilent feature extraction manual. The resulting intensities of the spots were used for the calculation of absolute difference and ratios for EAE vs. CFA control animals. The data analysis was performed using the Spotfire software for functional genomics, selecting genes by filtering on ratio and difference. We considered a gene up- or downregulated if the change in gene expression was visible in all four different hybridizations with a ratio EAE vs. CFA control of minimal 1.5. For further analysis, clinical scores were normalized. Minimum clinical scores were set at 0%, whereas maximum clinical scores were set at 100%. Similarly, fold changes in mRNA expression were normalized for all individual genes, with a minimum fold change in the course of EAE set at 0% and maximum fold change at 100%. Subsequently, relative least square differences (variance score Σ(clin. score − gen. score)2/clin. score) between normalized clinical scores and normalized fold changes were calculated for each gene. This parameter allowed filtering of data on the basis of variations in gene expression with respect to the clinical scores.

### Determination of anti-MOG IgG levels

The blood was drawn at 27 dpi, and antigen-specific serum antibody titers were measured by ELISA. Briefly, ELISA plates were coated with 10 μg/ml MOG_35–55_ in PBS, and the MOG-specific IgG serum antibody titer was measured using rabbit anti-mouse IgG HRP-linked antibody (DAKO) and biotinilated goat anti-mouse IgG1 and anti-mouse IgG2c (Jackson ImmunoResearch). The end point dilution was 2 times of the blank value.

### Brain tissue

In collaboration with The Netherlands Brain Bank (Amsterdam, The Netherlands, coordinator Dr. I. Huitinga), we used human post-mortem brain tissue from three non-neurological controls and eight MS patients (see [19] for patient details). The study was approved by the institutional ethics review board (VU University Medical Center, Amsterdam, The Netherlands), and all donors or their next of kin provided written informed consent for brain autopsy and use of material and clinical information for research purposes. Lesion types were determined by proteolipid protein (PLP) and MHC II staining (Additional file 1: Figure S1).

### Microglia isolation and culture

As described by Beaino et al. [19], briefly, 5 to 10 g of brain white matter was obtained at autopsy, and microglia isolation procedure was performed within 4 to 24 h. Single-cell suspensions were prepared using 0.05% trypsin (Sigma-Aldrich). Cells were then filtered through a 100-μm nylon mesh (BD Bioscience), centrifuged, and the pellet was resuspended in a gradient buffer (3.56 g/l Na_2_HPO_4_·2H_2_O, 0.78 g/l NaH_2_PO_4_·H_2_O, 8 g/l NaCl, 4 g/l KCl, 2 g/l d-(+)-glucose, and 2 g/l BSA, pH 7.4) and centrifuged for 35 min at 1200×*g*. The cell debri-myelin layer was removed. The cell pellet was treated with red blood cell lysis buffer (8.3 g/l NH_4_Cl and 1 g/l KHCO_3_, pH 7.4) for 10 min at 4 °C. Cells were then suspended and cultured in DMEM/Ham nutrient mixture F10 (1:1), 1% penicillin-streptomycin-glutamine, and 25 ng/ml granulocyte-macrophage colony-stimulating factor (GM-CSF is only added for the first 2 days). After 7 days, the microglia were polarized into M1 phenotype by priming with 1 × 10^3^ U/ml of rhIFN-γ (U-Cytech) for 24 h, followed by the addition of 10 ng/ml of *Escherichia coli* LPS (InvivoGen) to the medium for 24 h. M2 microglia were polarized by adding 10 ng/ml of IL-4 (Immunotools) for 48 h. Untreated cells are referred to as M0.

### Macrophage differentiation and culture

Human peripheral blood mononuclear cells were isolated from heparinized human peripheral blood from healthy donors by density gradient centrifugation on Lymphoprep™ (Stemcell Technologies), and monocytes were isolated using CD14-MACS microbeads (Miltenyi Biotec) according to the manufacturer’s protocol. All blood donors gave informed consent. Macrophages were generated by culturing monocytes for 5 days in RPMI 1640 medium (Invitrogen) containing 10% fetal calf serum, 2 mM glutamine 50 U/ml penicillin, and 50 μg/ml streptomycin (all from Lonza), in the presence of 50 ng/ml M-CSF (Miltenyi). On day 5, macrophages were polarized into M1 phenotype by priming with 50 ng/ml of rhIFN-γ (Peprotech) for 24 h, followed by the addition of 10 ng/ml of *E. coli* LPS (Sigma) to the medium for 24 h. M2 macrophages were polarized by adding 20 ng/ml of IL-4 (Immunotools) for 48 h. Untreated cells are referred to as M0. For MGL triggering experiments, macrophages (50.000) were plated on 96-well F-bottom plates coated with 1 μM Tn3−, gal-dendrimers, or uncoated wells in the presence or absence of 10 ng/ml LPS. Supernatants were harvested after 18 h, and IL-10 levels were determined by ELISA (eBioscience).

### Flow cytometry

For surface expression analysis, cells were incubated with primary antibodies according to the manufacturer’s instructions. For all flow data analyses, debris and dead cells were excluded by 7-AAD staining. Cells were acquired on a FACSCalibur™ (BD Biosciences) or on a CyAn ADP (Beckman Coulter) and analyzed with FlowJo software (Tree Star).

### MGL-binding assays

For analysis of MGL ligand expression, splenocytes or DLN cells were incubated for 30 min at 37 °C with MGL1− or MGL2-Fc (10 μg/ml) in a solution of 20 mM Tris-HCl, pH 7.4, 150 mM NaCl, 2 mM MgCl_2_, 1 mM CaCl_2_, and 0.5% (*w*/*v*) bovine serum albumin (BSA), followed by staining with secondary FITC-labeled anti-human Fc (Jackson ImmunoResearch) and analysis on a FACSCalibur.

### Immunohistochemistry and immunofluorescence staining

For human tissue staining, air-dried frozen sections (5 μm) were fixed with acetone (10 min at RT). For single immunohistochemistry staining of PLP, MHC-II, and MGL, we used the protocol described previously by Beaino et al. [19] using Envision-HRP (Dako) and 3,3′-diaminobenzidine (Dako) as a detection method. For immunofluorescence staining on human tissue, the sections were fixed with acetone then blocked for non-specific binding with goat serum (10%) for 20 min at RT. The sections were then incubated with the primary antibody in PBS/1% serum overnight at 4 °C. The sections were then washed with PBS three times for 5 min and incubated with fluorescently labeled secondary antibody in PBS/1% serum or for 1 h at RT. The nuclei were stained with Hoechst (1/1000) for 1 min in the dark. After a final wash for three times for 5 min with PBS, the sections were mounted with coverslips using aqueous mounting media Mowiol.

### Real-time quantitative RT-PCR

Total mRNA from polarized microglia was extracted using the mRNA Capture Kit (Roche), and cDNA was synthesized using the Reverse Transcription System kit (Promega) following the manufacturer’s guidelines. Reactions were performed using the SYBR Green method in an ABI 7900HT sequence detection system (Applied Biosystems), with GAPDH as the endogenous reference gene (ERG). Samples were analyzed in duplicate and normalized to GAPDH. Oligonucleotides were designed by using the computer software Primer Express 2.0 (Applied Biosystems) and synthesized by Invitrogen. Primer specificity was computer tested by homology search with the human genome (BLAST, National Center for Biotechnology Information) and later confirmed by dissociation curve analysis. The difference between the Ct of the target gene and the Ct of the ERG ΔCt = Ct_Target_ − Ct_ERG_ is used to obtain the normalized amount of target (Nt), which corresponds to 2^−ΔCt^. The Nt reflects the relative amount of target transcripts with respect to the expression of the ERG. Primer sequences: CLEC10A, Fw, 5′-TACACCTGGATGGGCCTCAG-3′, Rev., 5′-TGTTCCATCCACCCACTTCC-3′; GAPDH, Fw 5′-CCTTCCGTGTCCCCACTG-3′, rev 5′-GACGCCTGCTTCACCACC-3′.

### Dataset selection and analysis

Expression of MGL (CLEC10A) in a RNA-Seq data previously published by Hendrickx et al. (GSE108000) [20] was analyzed using the R-based software RStudio. Normalized data was downloaded using the package *GEOquery* [21] and converted to *z*-score using the function *scale*.

### Statistical analysis

Prism7 software (GraphPad) was used for statistical analysis. For clinical EAE and weight data, the area under the curve (AUC) was calculated for each mouse over the time period assessed, and these values were used for comparison of groups by an unpaired, one-tailed Student’s *t* test. Statistical differences in the mean maximum scores were analyzed by one-tailed Mann-Whitney *U* test. Differences with *P* values of less than 0.05 were considered significant.

## Results

### MGL expression in autoimmune neuroinflammation

**Fig. 1:**
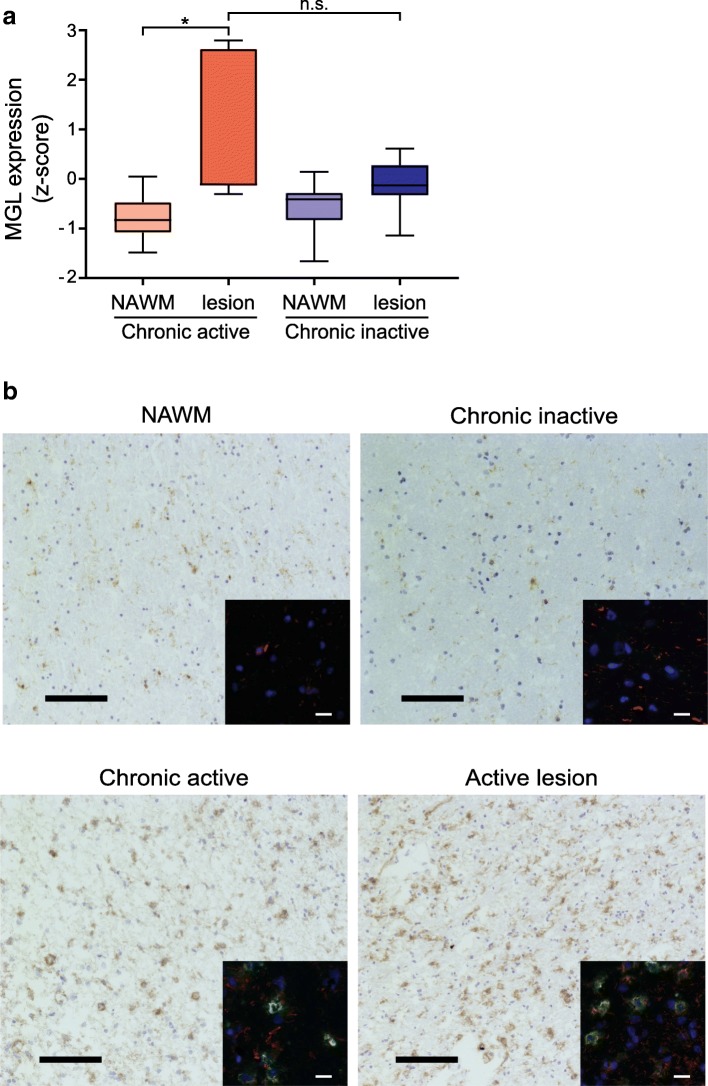
MGL expression is upregulated in MS lesions. **a** Box plot showing the *z*-transformed expression values of MGL in NAWM and rim from chronic inactive (*n* = 7) and active (*n* = 7) MS lesions. *P < 0.05 (Kruskal-Wallis test followed by Dunn’s multiple comparisons test). **b** Expression of MGL in normal-appearing white matter (NAWM) and MS chronic inactive, chronic active, and active lesions. Consecutive brain sections from MS patients were stained for MGL and imaged by standard microscopy (representative of 3 donors). Scale bars, 50 μm. Insets, double staining showing the expression of MGL (red) and MHC II (green). Blue is a nuclear staining with DAPI. Images were collected from the same section with the same exposure time between different areas to allow comparison. Scale bars, 15 μm

MGL has been shown to have immunoregulatory properties when expressed on tolerogenic APCs both in autoimmune and cancer settings [8–13]. However, its role in autoimmune neuroinflammation has not been reported. Thus, we wanted to explore whether MGL plays a role in MS. To that end, using publicly available data [20], we compared the expression levels of the MGL gene, CLEC10A, in normal-appearing white matter (NAWM) and rim sections of chronic active and inactive MS lesions. Fully active demyelination occurs in the rim of chronic active MS lesions, while the rim of inactive MS lesions denotes halt of demyelination [20]. Notably, the rim of active lesions expressed higher levels of MGL mRNA compared to their NAWM sections. Moreover, we found the same low levels of MGL mRNA in NAWM and rim sections of chronic inactive MS lesions, and although not significant, *z*-score values showed a clear difference in MGL expression, with higher expression of this lectin in the rim of active lesions compared to those from inactive lesions (Fig. 1a), suggesting a role for this endogenous lectin in areas of neuroinflammation. To validate these results, we investigated the expression levels of MGL in chronic active, chronic inactive, and active MS lesions by IHC. Confirming the in silico data, MGL was weakly expressed or absent in NAWM or chronic inactive lesion of MS patients, while its expression was markedly upregulated in the active and chronic active lesions of MS patients (Fig. 1b and Additional file 1: Figure S1). Remarkably, MGL was expressed by foamy macrophages and activated microglia (Fig. 1b), and consistently, the expression of MGL co-localized with MHC II, demonstrating that only APCs express this CLR (Fig. 1b, insets). Of note, foamy macrophages are alternatively activated APCs that express anti-inflammatory cytokines and lack typical pro-inflammatory cytokines [22].

Importantly, in a rat acute monophasic EAE model, MGL was also highly expressed at the peak of the clinical disease (Additional file 1: Figure S2). These data indicate that the expression of human (Fig. 1a, b) or rat MGL (Additional file 1: Figure S2) is upregulated under inflammatory conditions in the brain. Moreover, their expression pattern, similar to those of the endogenous immune inhibitory molecules PD-1 and CTLA-4 [23, 24], further indicates that MGL may participate in limiting the inflammatory response, thus contributing to disease recovery.

### MGL is expressed on alternatively activated microglia

**Fig. 2:**
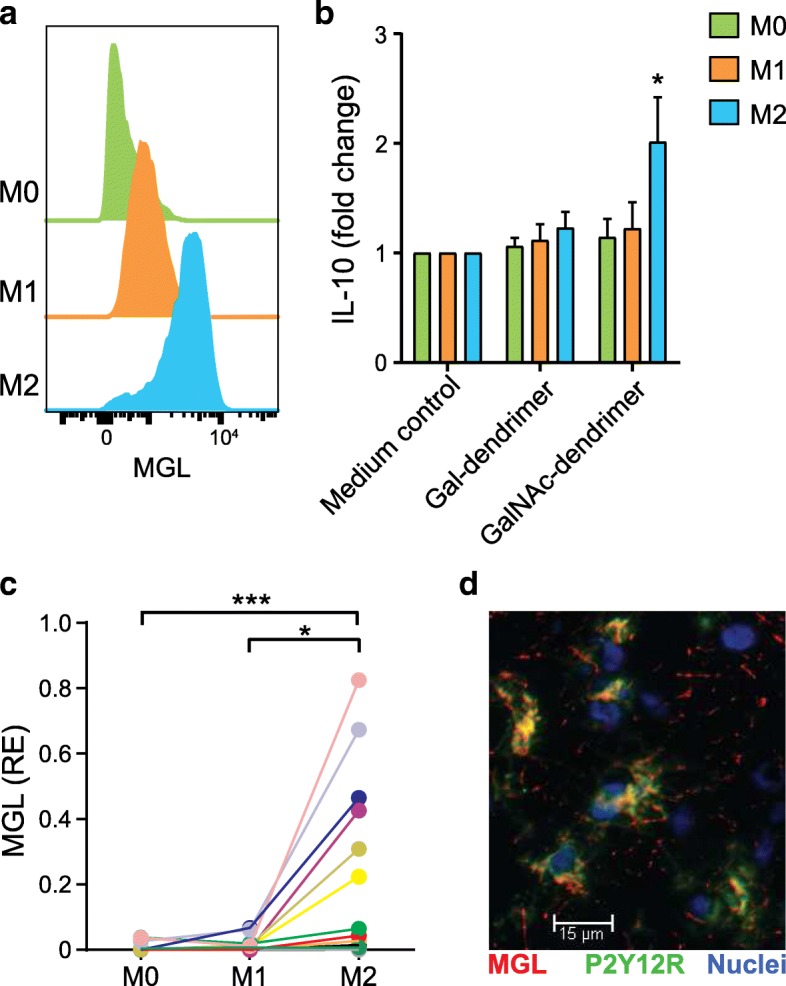
MGL is expressed on alternatively activated microglia. **a**, **b** Monocyte-derived macrophages were polarized to the M0, M1, and M2 phenotypes. **a** Flow cytometry histograms. Representative plots from three independent experiments. **b** M0, M1, and M2 macrophages were stimulated with 10 ng/ml LPS in the presence or absence of GalNAc dendrimer (MGL agonist) or Gal-dendrimer (control dendrimer). After ON incubation, IL-10 was measured in the supernatants by ELISA. Values (fold change) are relative to that of control medium (medium without dendrimers). Mean + SEM, Mann Whitney *U* test, *P < 0.05 vs control treatment. Data are from two independent experiments with cells from four different donors. **c** Primary human microglia was isolated from freshly obtained post-mortem brain tissue and further polarized to the M0, M1, and M2 phenotypes. Cells were then lysed for mRNA isolation and the expression levels of MGL assayed by real-time quantitative RT-PCR. Each color identifies the cells from one donor. Values (relative expression, RE) are relative to that of GAPDH mRNA. *P < 0.05; ***P < 0.001 (Friedman test followed by Dunn’s multiple comparisons test). **d** Confocal microscopy in MS active lesion showing the merged expression of MGL (red) and P2Y12R (green). Nuclear staining (blue). Images were collected from the same section as in Fig. 1b. Scale bar, 15 μm

It has been previously shown that macrophages express MGL [5, 25]; however, IL-10 secretion induced by human MGL has been demonstrated only in tolerogenic or immature DCs [5, 12, 13]. Given that we found MGL expression by foamy macrophages and/or activated microglia (Fig. 1b), we wondered whether this expression was specific of alternatively activated (M2) cells and whether MGL binding triggers IL-10 secretion by macrophage-type cells. We first differentiated monocytes in vitro to macrophages (M0) and then further activated them to a pro-inflammatory (M1)- or to an alternatively activated or anti-inflammatory (M2)-type macrophage and measured MGL expression. Confirming previous findings [25], only M2 macrophages expressed high levels of MGL (Fig. 2a). Next, for functional analysis of the receptor, we incubated the different polarized macrophages in the presence or absence of the multivalent MGL ligand GalNAc dendrimer (MGL agonist) or its control (gal-dendrimer). Notably, only M2 cells showed an enhanced secretion of IL-10 (Fig. 2b), similar to what was found in tolerogenic MGL-expressing DCs [12, 13]. Of note, this is the first evidence demonstrating the functionality of MGL triggering on human M2 macrophages.

The microglia, the resident phagocytic cells in the brain, can also be oversimplified classified as M1 and M2 cells, with detrimental and beneficial effects on MS, respectively [26, 27]. Due to the pivotal role of these cells in CNS inflammation and their role as APCs [28, 29], we studied the expression of MGL on human microglia cells. For this, we isolated human microglia (M0) and further polarized them to M1 and M2 phenotype as we described previously [19]. Similar to what we found in in vitro differentiated macrophages, MGL was expressed at higher levels in microglia polarized into the M2 phenotype when compared to M0- or M1-polarized microglia (Fig. 2c). Importantly, in MS active lesions, MGL protein was expressed by M2-polarized cells as MGL co-localized with the M2 macrophage/microglia marker P2Y12R (Fig. 2d) [19], further indicating a link between M2 phenotype and MGL expression. Altogether, these data demonstrate MGL tissue localization during CNS inflammation as well as expression and functionality is restricted to M2 cells, together supporting the hypothesis that this endogenous lectin may participate as a negative regulator in MS.

### MGL1-deficient mice are highly susceptible to EAE

**Fig. 3:**
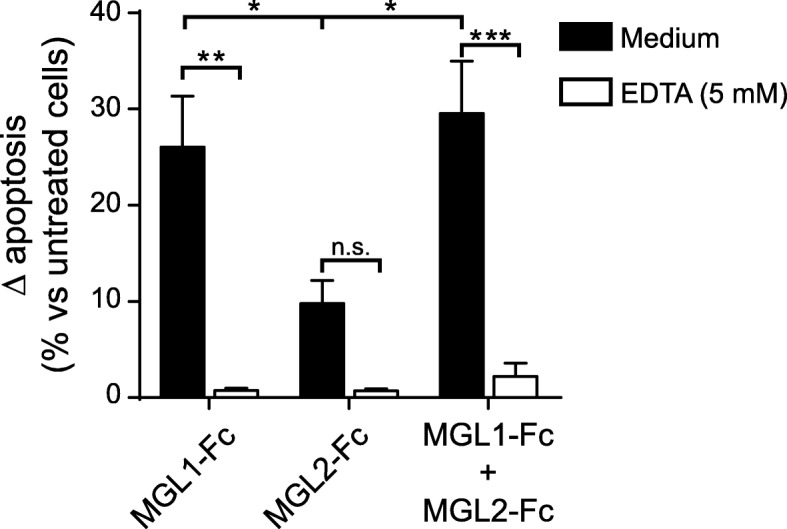
MGL1 but not MGL2 induces apoptosis of activated mouse T helper cells. Naïve CD4^+^ T cells from wild-type C57Bl/6 mice were stimulated for 3 days with CD3 and CD28 mAbs. Activated cells were washed and left untreated or incubated ON with 10 μg/ml of the indicated Fc-chimeras in the presence (white bars) or absence (black bars) of the Ca^2+^-chelator EDTA (5 mM). Then, cells were washed and stained with 7-AAD and AV and analyzed by flow cytometry. Cell death was determined as the difference in the percentage of AV^+^ cells between polarized cells treated with or without MGL-Fcs. Data are from three independent experiments with cells from three mice. One-way ANOVA followed by Tukey’s multiple comparisons test; *P < 0.05; **P < 0.01; ***P < 0.001

We next investigated the relevance of MGL in an in vivo model of autoimmune neuroinflammation. Given the non-redundant functions of the murine MGL1 and MGL2 homologs and that they both share functions with their human ortholog [5], we first investigated whether the murine MGLs were able of inducing apoptosis in effector CD4^+^ T cells. Previously, we have shown in human in vitro assays that MGL expressed on tolerogenic DCs binds to CD45 on activated CD4^+^ cells thereby reducing T cell proliferation and cytokine production and leading to T cell apoptosis [8]. Nevertheless, a similar mechanism has not yet been reported in mice. We activated naïve CD4^+^ cells in vitro and exposed them ON to 10 μg/ml MGL1− and/or MGL2-Fc chimeras in the presence or absence of the Ca^2+^-chelator (acting as a C-type lectin inhibitor) ethylenediaminetetraacetic acid (EDTA). As shown in Fig. 3, MGL1, but not MGL2, induced apoptosis of activated T helper cells. This effect was carbohydrate specific, as the addition of EDTA inhibited MGL1-induced apoptosis (Fig. 3). These data show for the first time that the binding of MGL1 to mouse effector CD4^+^ T cells is sufficient to induce their apoptosis.

**Fig. 4:**
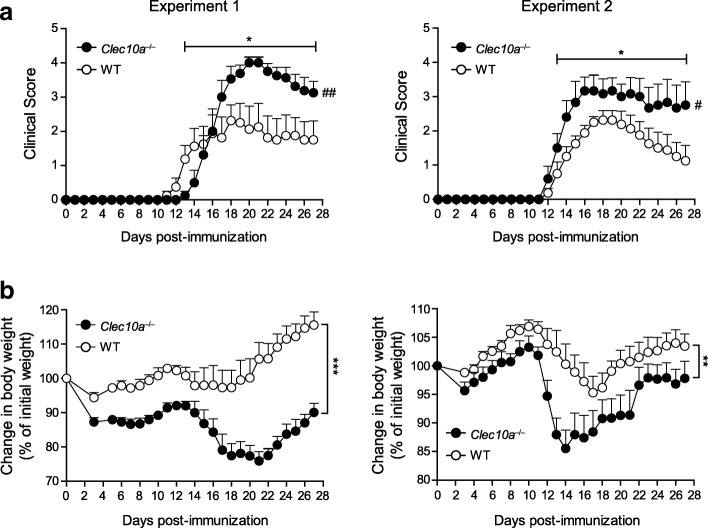
MGL1 deficiency results in enhanced susceptibility to EAE. Wild-type (WT) and *Clec10a*^−/−^ mice were immunized with MOG_35–55_ and examined for disease progression (**a**) and body weight changes (**b**). Two independent experiments with 6–8 mice per group are shown. Values represent the mean + SEM; *P < 0.05; **P < 0.01; ***P < 0.001 (one-tailed unpaired Student’s *t* test of the AUC); ^#^*P* < 0.05; ^##^*P* < 0.01 (one-tailed Mann-Whitney of the mean maximum scores)

The increased CD4^+^ cell apoptosis induced by MGL1 prompted us to investigate whether endogenous MGL1 has a relevant pathophysiological function on the development and progression of CNS inflammation in EAE, a model whose pathogenicity is mediated by autoreactive CD4^+^ T cells [30]. We immunized MGL1-knockout (*Clec10a*^−/−^) and wild-type (WT) C57BL/6 mice with the encephalitogenic peptide (amino acids 35–55) of myelin oligodendrocyte glycoprotein (MOG_35–55_). Strikingly, although the overall EAE incidence and time of disease onset were similar between the two groups, disease severity and weight loss were much greater in *Clec10a*^−/−^ mice than in their WT littermates (Fig. 4a, b), suggesting that MGL1 is able to halt autoimmune neuroinflammation. Moreover, this is the first report demonstrating that MGL1 is involved in EAE susceptibility.

### Mice devoid of MGL1 show an increased MOG-specific response

**Fig. 5:**
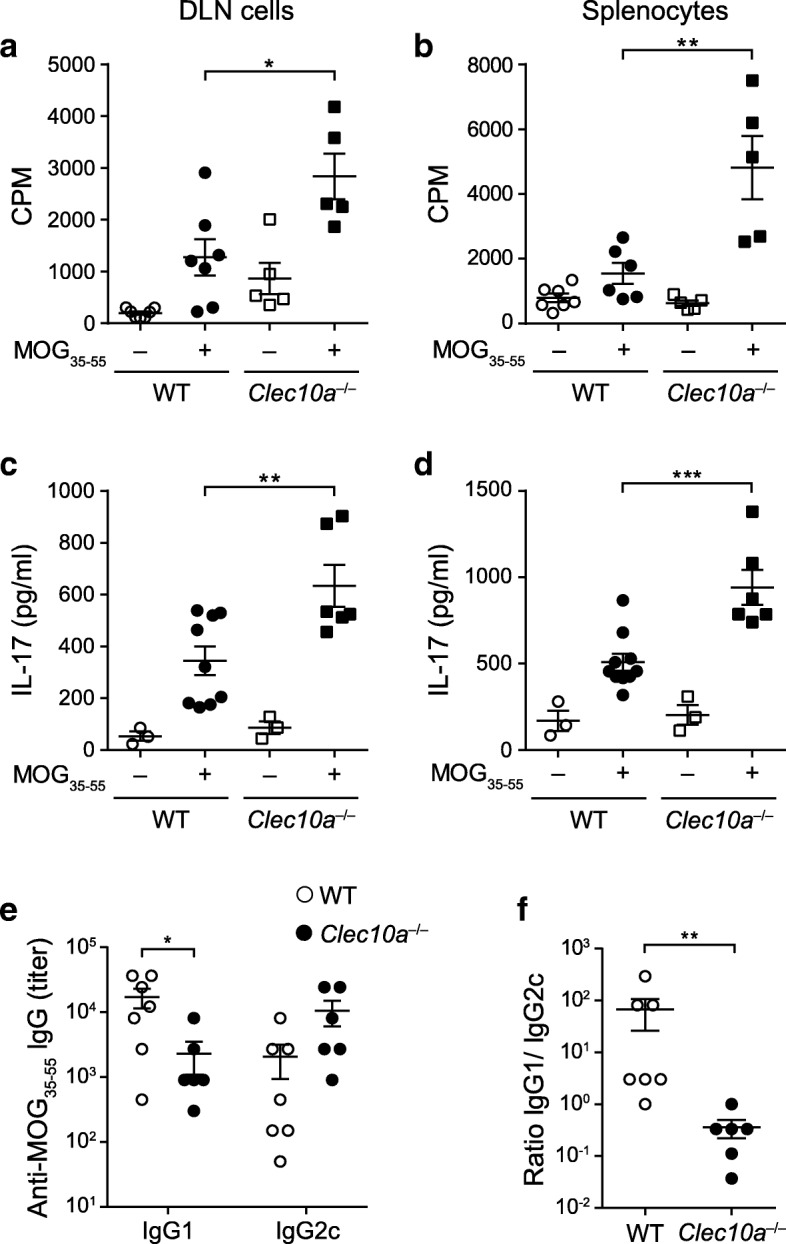
Increased pro-inflammatory immune response in *Clec10a*^−/−^ mice. MOG_35–55_-specific proliferation (**a**, **b**) and IL-17 production (**c**, **d**) by DLN cells (**a**, **c**) and splenocytes (**b**, **d**) from WT and *Clec10*^−/−^ mice 27 days post-immunization (dpi; endpoint of experiments in Fig. 2). Proliferation was assessed by [^3^H]-thymidine incorporation, and cytokine secretion was analyzed by ELISA after 72 h of MOG_35–55_ restimulation. Values represent the mean ± SEM; *P < 0.05; **P < 0.01; ***P < 0.001 vs. cells from WT mice stimulated with MOG_35–55_ (Student *t* test). **e** Serum anti-MOG_35–55_ IgG1 and IgG2a antibody titers from WT and *Clec10*^−/−^ mice obtained at 27 dpi. Mean ± SEM. Mann Whitney *U* test, *P < 0.05. **f** IgG1/IgG2c ratio per mouse from the titers shown in **e**. Mean ± SEM. Mann Whitney *U* test, **P < 0.01. **a**–**f** One representative experiment out of two is shown

To understand the mechanistic basis of the exacerbated EAE in *Clec10a*^−/−^ mice, we first evaluated the MOG_35–55_-specific proliferation and cytokine production of draining lymph node (DLN) mononuclear cells (draining sites of immunization) and splenocytes from *Clec10a*^−/−^ and WT mice, as autoreactive T cells play a pivotal role in the disease severity of MS and EAE [30]. The EAE pace in *Clec10a*^−/−^ mice, similar to other well-described immune inhibitory pathways involved in EAE amelioration such as PD-L1 [24] and galectin-1 [31] suggested that the mechanisms mediated by MGL1 should be still active at 27 dpi, contributing to the resolution of the inflammation. Accordingly, at this time point, cells from *Clec10a*^−/−^ mice showed a more vigorous antigen-specific proliferation compared to cells derived from WT littermates, when restimulated ex vivo with MOG_35–55_ (Fig. 5a, b). Furthermore, DLN cells and splenocytes from *Clec10a*^−/−^ mice produced more IL-17 after antigen restimulation than their WT counterparts (Fig. 5c, d). However, we found no substantial differences in the production of IFN-γ or IL-10 between *Clec10a*^−/−^ and WT cells (data not shown).

Given the relevant pathogenicity of B cells in MS [32, 33], we next studied the titers and isotypes of the anti-MOG antibodies. Notably, the heightened inflammatory response observed in *Clec10a*^−/−^ T cells was also evident in the B cell compartment. Although the amount of total anti-MOG IgG was comparable between WT and *Clec10a*^−/−^ mice (data not shown), the IgG1/IgG2c ratio of MOG-specific antibodies was more than 100 times higher in serum from WT mice than in their KO counterpart (Fig. 5e, f), indicating that the type of humoral response in MGL knockout mice switched from a predominantly Th2 response, with the production of IgG1 antibodies, to a more pro-inflammatory response, with the production of IgG2c antibodies [34–36]. Collectively, these findings indicate that MGL1 negatively regulates the CNS inflammatory response by altering the production of autoantibodies and dampening of the autoreactive T cell response.

### MGL1 induces apoptosis of effector T cells

**Fig. 6:**
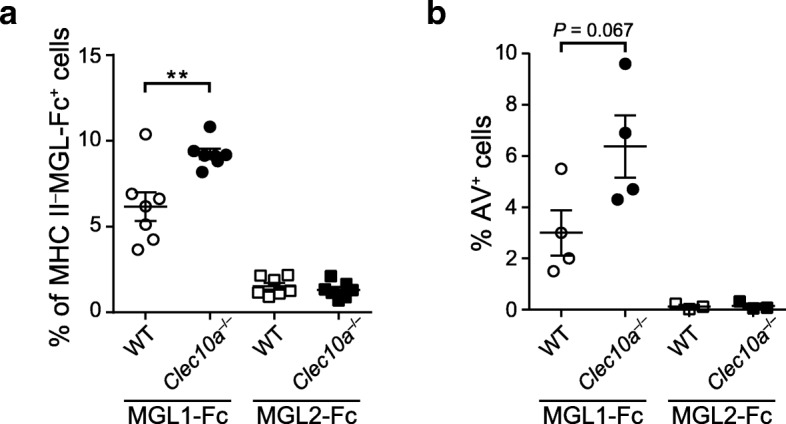
Cells from *Clec10a*^−/−^ mice are more susceptible to MGL1-induced cell death. **a**, **b** Wild-type (WT) and MGL1-deficient (*Clec10a*^−/−^) mice were immunized with MOG_35–55_ for EAE induction. At 27 dpi, DLN cells were isolated and incubated in the presence or absence of MGL1-Fc or MGL2-Fc. **a** After 30 min of incubation, cells were stained with anti-human IgG Fc to determine MGL1/2 binding and gated on MHC II^−^ cells. **b** Cell death was evaluated by AV binding after ON incubation. Mean ± SEM. Student *t* test, **P < 0.01

Given the pro-apoptotic effect of MGL1 in vitro (Fig. 3), we next studied whether the programmed cell death induced by this endogenous lectin was responsible for the lower clinical score and the lower inflammatory MOG-specific immune response observed in WT mice. For this, at day 27 after EAE induction, we isolated DLN cells from WT and *Clec10a*^−/−^ mice and incubated them in the presence or absence of MGL1-Fc and MGL2-Fc. We observed a higher binding of MGL1-Fc to lymphocytes from *Clec10a*^−/−^ mice than lymphocytes from their WT counterpart (Fig. 6a) and that this binding correlated with higher apoptosis induction (Fig. 6b). These results suggest that, in the absence of MGL1 in vivo, fewer activated T cells are undergoing apoptosis leading to an increased pathology. In correlation with its lack of binding, MGL2-Fc did not induce apoptosis (Fig. 6b, c). Thus, deletion of MGL1 resulted in exacerbated MOG-specific immune responses, demonstrating a critical function for MGL1 in dampening pathogenic T and B cell responses in vivo, an effect that possibly relates to the lack of apoptosis of effector T cells in mice devoid of MGL1.

## Discussion

Human MGL is expressed on immature and tolerogenic DCs, dermal CD1a^+^ DCs, and blood CD1c^+^ myeloid DCs [5]. Macrophages express MGL; however, it is especially upregulated on alternatively activated macrophages [5, 37]. Human MGL and murine MGL1 and MGL2 have immunoregulatory properties when expressed on tolerogenic APCs and have been shown to play a role in ameliorating autoimmune diseases [5, 7, 10–13, 15, 16]. It has been shown that MGL is expressed by APCs at sites of chronic inflammation in rheumatoid arthritis [8]. Here, we found that MGL is markedly expressed at sites of neuroinflammation. By microscopy and tissue microarray analysis, we observed that MGL is highly expressed in active and chronic active MS lesions, but not in chronic inactive MS lesions or NAWM (Fig. 1a, b), indicating an attempt to prevent lesion expansion and progression, as has been suggested for other anti-inflammatory genes upregulated in chronic active MS lesions [20]. In line with these findings, we demonstrate for the first time that *Clec10a*^−/−^ mice show an exacerbated EAE disease severity (Fig. 4a), indicating a role for MGL1 in limiting CNS inflammation.

Expression patterns of immune checkpoint inhibitors in EAE indicate that their levels rise after disease onset, reach a plateau at the peak of the disease, and remain stable or diminish during the resolution phase of the inflammation [23, 24, 31]. Moreover, the pace of the EAE in the absence of immune checkpoint inhibitors shows a similar disease onset but higher scoring when compared to WT controls [31, 38–41]. In line with this, we have shown that human MGL and rat MGL are increased in the CNS during MS or EAE inflammation. In rat EAE, MGL is upregulated at the peak of the disease in both cerebellum and brainstem and then diminishes when inflammation is resolved (Additional file 1: Figure S2). Moreover, EAE in *Clec10a*^−/−^ mice exhibits a similar disease onset, but higher disease severity [31, 38–41], suggesting that MGL1 might act as an immune checkpoint inhibitor in CNS autoimmune inflammation. Future research will address whether, similarly to human MS lesions and acute EAE in rats, MGL1 is upregulated at the peak and/or resolution phase of the EAE in mice. Furthermore, given the above-indicated link between MGL1 and IL-10 and the pivotal role of IL-10 in EAE resolution (ref. [42, 43]) more work is needed to determine whether, as in human M2 microglia (Fig. 2b), MGL1 binding in murine CNS APCs induces the secretion of IL-10.

The mechanisms underlying the anti-inflammatory activity of MGL1 remain poorly understood. Human MGL inhibits DC maturation and migration, increases their IL-10 secretion [9–14], and induces CD45-dependent CD4^+^ T cell apoptosis, thereby dampening the inflammatory response [8]. Available information about the role of MGL1 in autoimmune disease models is limited but suggestive of an anti-inflammatory role. In dextran sulfate sodium salt-induced colitis, intestinal lamina propria macrophages expressing MGL1 increase IL-10 production in response to commensal *Streptococcus* bacteria, playing a protective role against autoimmune colitis [10]. In this work, we show for the first time that MGL is also upregulated on M2 microglia, cells that show neuroprotective and regenerative properties in the CNS [27, 44], co-localizing with the M2 macrophage/microglia marker P2Y12R (Fig. 2c, d) [19]. At the same time, we demonstrate that M2 macrophages express MGL and, like MGL-expressing DCs [13], produce IL-10 in response to stimulation with MGL-specific ligands (Fig. 2a, b). Further research has to address whether MGL triggering on human M2 microglia cells induces IL-10.

Apoptosis induction of antigen-specific T cells is the mechanism of action shared by the checkpoint inhibitors PD-1, Galectin-1, and Tim-3 [38, 41, 45]. Mouse MGL1 and MGL2 possess non-redundant roles; however, the functions described for each one fulfils most of the human MGL characteristics (reviewed in [5]). Here, we show that MGL1, but not MGL2, induces apoptosis of activated CD4^+^ T cells (Fig. 6b). Moreover, ex vivo experiments show that mononuclear MHC II^−^ DLN cells from MGL1-devoid mice with EAE bind stronger to MGL1, correlating with higher MGL1-induced apoptosis, when compared to cells from WT mice (Fig. 6a, b). Whether MGL1 is inducing apoptosis of CD4^+^ T cells or of a different MHC II^−^ lymphocyte subpopulation in vivo still needs to be investigated. In this sense, and given that autoreactive T cells play a crucial role in the disease severity of MS and EAE, the augmented EAE severity in *Clec10a*^−/−^ mice observed could be the result of an increased number of MOG_35–55_-specific effector T cells. Accordingly, splenocytes and DLN cells from MGL1-devoid mice showed higher proliferation and secreted more IL-17 than their WT counterpart (Fig. 5a–d). Intriguingly, we found no differences in IL-10 production. This was unexpected given the central role of IL-10 in the resolution of autoimmune neuroinflammation [42, 46] and the previously described MGL1-dependent IL-10 secretion mechanisms [5, 10]. Whether these mechanisms are also driven by MGL^+^ cells in MS requires further research. Altogether, these findings indicate that MGL M2 microglia could play a role in resolving neuroinflammation by increasing IL-10 production and by inducing apoptosis in autoreactive CD4^+^ T cells. Moreover, we have uncovered a novel marker for M2 microglia identification.

Human MGL or mouse MGL1 are not expressed on B cells and have not been previously associated with the alteration of the B cell response. Nevertheless, we have found that the humoral response in *Clec10a*^−/−^ mice switched to a predominant Th1 response, evidenced by the high titer of IgG2c isotype in comparison with higher levels of the more anti-inflammatory Th2 isotype IgG1 in the serum of WT mice (Fig. 5e, f) [34–36]. B cells at the B cell follicle form the germinal center under the influence of specialized T follicular helper (T_FH_) cells [47]. T_FH_ cells collaborate with B cell proliferation and class-switch recombination by the expression of CD40L and cytokine production [47]. Whether MGL1^+^ dendritic cells or macrophages migrate to secondary lymphoid organs and affect T follicular helper cells or the cytokine expression at the germinal center microenvironment remains to be explored.

Successful treatment of MS should aim at two different processes in order to achieve a complete remission: (1) to abrogate disease progression and (2) to stimulate the resolution of inflammation. However, just a few ligand-receptor axes have been described as fulfilling one or both of these requirements. So far, CTLA-4–CD80/86, PD-1–PD-L1/2, TIGIT–CD112/155, TIM-3–galectin-9, and CD43/45–galectin-1 are the only immune inhibitory pathways involved in EAE amelioration with a strong genetic or functional correlation in MS [4, 24, 31, 38–41, 48]. Here, we show that the endogenous lectin MGL tackles both targets. MGL is highly expressed at sites of neuroinflammation and triggers IL-10 production by macrophages that in turn might collaborate to the resolution of the disease. At the same time, MGL binding to its ligand CD45 on CD4^+^ T cells induces apoptosis of effector T cells, thereby inhibiting disease progression. Altogether, these results provide for the first time evidence that both MGL- and MGL1-expressing APCs might play a role in limiting the inflammatory response by fostering an anti-inflammatory environment in MS or EAE by increasing IL-10 secretion and inducing apoptosis of effector T cells.

## Conclusion

The data presented in this study indicate a role of MGL as a negative regulator of CNS autoimmune inflammation and a marker for human M2 microglia. The confirmation of this role by future investigations will permit the study of this endogenous lectin as a knew immunotherapeutic tool in MS and other autoimmune conditions.

## Additional file

Additional file 1: **Figure S1.** PLP and MHC-II staining in different MS lesions and control sections. Figure S2. MGL expression during rat acute EAE. (PDF 398 kb)

## Data Availability

The datasets used and/or analyzed during the current study are available from the corresponding author on reasonable request.

## Abbreviations

ANOVA: One-way analysis of variance
APC: Antigen-presenting cell
CFA: Complete Freund’s adjuvant
CNS: Central nervous system
DC: Dendritic cell
EAE: Experimental autoimmune encephalomyelitis
EDTA: Ethylenediaminetetraacetic acid
FITC: Fluorescein isothiocyanate
GM-CSF: Granulocyte-macrophage colony-stimulating factor
M-CSF: Macrophage colony-stimulating factor
MGL: Macrophage galactose-type lectin
MS: Multiple sclerosis
NAWM: Normal-appearing white matter
PE: Phycoerythrin
qPCR: Quantitative real-time polymerase chain reaction
RT: Room temperature
SD: Standard deviation
SEM: Standard error of the mean
T_FH_: T follicular helper
