# Supplementary material for: Macrophage galactose-type lectin (MGL) is induced on M2 microglia and participates in the resolution phase of autoimmune neuroinflammation
Source: J Neuroinflammation. 2019 Jun 27;16:130. doi: 10.1186/s12974-019-1522-4 (PMC6598247; doi:10.1186/s12974-019-1522-4)
Supplement: Supplementary file 1 — Figure S1. PLP and MHC-II staining in different MS lesions and control sections. Figure S2. MGL expression during rat acute EAE. (PDF 398 kb) [file 12974_2019_1522_MOESM1_ESM.pdf]

Additional Figures

Macrophage galactose-type lectin (MGL) is induced on M2 microglia and participates in the resolution phase of autoimmune neuroinflammation

Juan M. Ilarregui, Gijs Kooij, Ernesto Rodríguez, Susanne M. A. van der Pol, Nathalie Koning, Joost C. van der Horst, Sandra J. van Vliet, Juan J. García-Vallejo, Helga E. de Vries & Yvette van Kooyk.

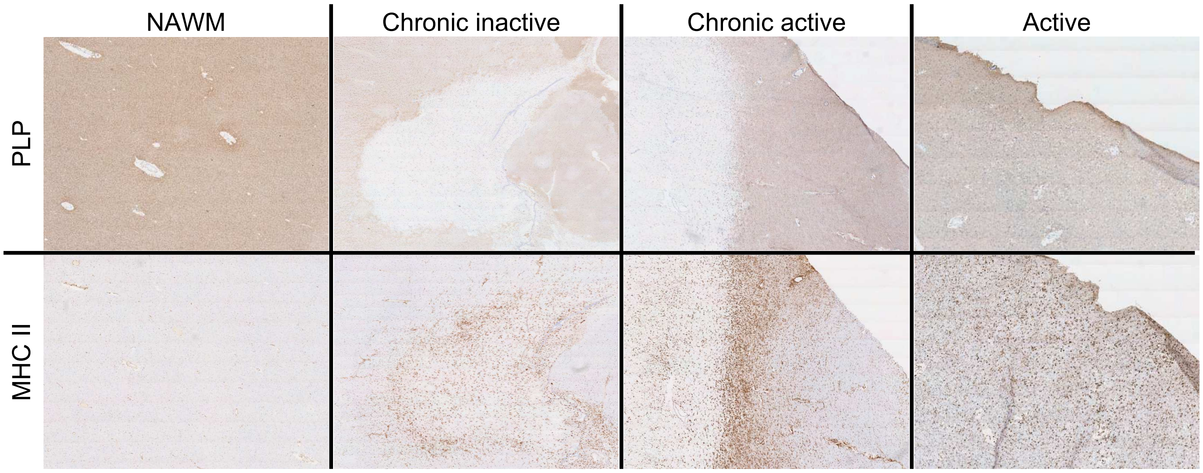

Additional file 1: Figure S1. PLP and MHC-II staining in different MS lesions and control sections. PLP and MHC II immunohistochemistry from sections used at Fig. 1a showing NAWM, MS active, chronic active, and chronic inactive lesions. Magnification, ×20.

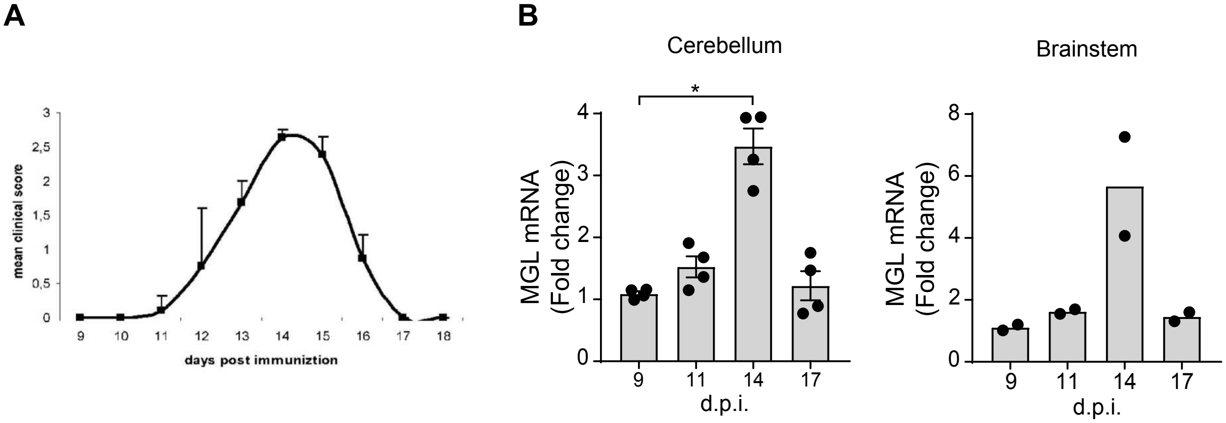

Additional file 1: Figure S2. MGL expression during rat acute EAE. (**a**) Time course of clinical scores in acute rat EAE. Values represent the mean + s.e.m of one experiment (n = 14). (**b**) MGL mRNA expression in the cerebellum (center) and brain stem (right) before onset of clinical disease (day 9), at the onset of the disease (day 11), at the peak of the disease (day 14) and after recovery (day 17) compared to RNA expression to that of CFA control animals. ***, $P < 0.001$ (one-way ANOVA followed by Tukey's multiple comparisons test).
